# Supplementary material for: Decline in Iran’s groundwater recharge
Source: Nat Commun. 2023 Oct 21;14:6674. doi: 10.1038/s41467-023-42411-2 (PMC10590393; doi:10.1038/s41467-023-42411-2)
Supplement: Supplementary file 1 — Supplementary Information [file 41467_2023_42411_MOESM1_ESM.pdf]

SUPPLEMENTARY MATERIALS FOR:

Decline in Iran's Groundwater Recharge

Roohollah Noori[1,2,*], Mohsen Maghrebi[1], Søren Jessen[3], Sayed M. Bateni[4], Essam Heggy[5,6], Saman Javadi[7], Mojtaba Noury[8], Severin Pistre[9], Soroush Abolfathi[10], Amir AghaKouchak[11,12,13]

[1]Graduate Faculty of Environment, University of Tehran, Tehran, Iran.

[2]Faculty of Governance, University of Tehran, Tehran, Iran.

[3]Department of Geosciences and Natural Resource Management, University of Copenhagen, Copenhagen, Denmark.

[4]Department of Civil and Environmental Engineering and Water Resources Research Center, University of Hawaii at Manoa, Honolulu, HI, USA.

[5]Viterbi School of Engineering, University of Southern California, Los Angeles, CA, USA.

[6]Jet Propulsion Laboratory, California Institute of Technology, Pasadena, CA, USA.

[7]Department of Water Engineering, College of Abouraihan, University of Tehran, Tehran, Iran.

[8]Iran Water Resources Management Company, Ministry of Energy, Tehran, Iran.

[9]HydroSciences Montpellier, University of Montpellier, CNRS, IRD, Montpellier, France.

[10]School of Engineering, University of Warwick, Coventry, UK.

[11]Department of Civil and Environmental Engineering, University of California, Irvine, USA.

[12]Department of Earth System Science, University of California, Irvine, USA.

[13]Institute for Water, Environment and Health, United Nations University, Hamilton, ON, Canada.

[*]Corresponding author:
Roohollah Noori (noor@ut.ac.ir); ORCID: http://orcid.org/0000-0002-7463-8563

Contents of this file

Notes S1 to S3

References

Figures S1 to S5

Note S1

Groundwater resources play a vital role in meeting the annual water demands for human life in Iran, with their contribution reaching as high as 90% in certain arid regions predominantly situated in the central and eastern parts of the country. Iranian aquifers annually supply about 40 km^3 renewable water to support the country's water demands during normal rainy years. However, unsustainable development in the shadow of climate change has resulted in severe stress to the country's groundwater resources, with an annual overexploitation of about 5-6 billion cubic meter[1]. This has led to the use of about 140 km^3 of non-renewable water from the country's aquifers by 2022[2]. Meanwhile, a report presented to the Academy of Sciences of the Islamic Republic of Iran raised more concerns regarding the declined recharge and excessive use of non-renewable water from the country's aquifers causing catastrophic and irreversible damage to the health of Iran's sub-surface water resources. This report claimed that the annual overexploitation of aquifers is approximately 15.9 km^3 and the country has lost around 350 out of 500 km^3 of its non-renewable groundwater resources. This means Iran's fresh groundwater resources will completely deplete by the early 2030s.

The tragedy of the demise of the country's aquifers and its dire consequences has now become a significant concern nationwide and internationally. To replenish the depleted groundwater resources, Iran's is endeavoring to compelet a national action plan that aims to artificially recharge aquifers up to 950 million cubic meter (MCM), Firouz Ghasemzadeh, the spokesperson for the Water Sector of Iran's Energy Ministry, stated. In this plan, 318 artificial recharge projects with a capacity of 700 MCM have been implemented throughout the country. In addition, 96 recharge projetcs with a total capacity of 145 MCM are in operation or under planning (https://www.farsnews.ir/news/14020420000736). However, this recharge volume (around 1 km^3) is far less than the defecit in the country's groundwater resources, particularly in areas where other factors, such as countrywide land subsidence, could hinder the effectiveness of recharge efforts. Iran is ranked among the top three countries with the highest rate of land subsidence, primarily caused by the over-abstraction of groundwater resources (https://financialtribune.com/articles/energy/113593). One of the largest land subsidence events in the country has occurred in Kashan city, starting near this city and extending all the way to the city of Qom, covering a distance of approximately 76 kilometer[3]. Also, a 20 m deep sinkhole with a diameter of 80 m has recently appread in Eaj, a city located in south east of Shiraz[4]. All provinces of Iran are now subject to land subsidence (except Guilan province), Ali Javidaneh, the General Manager for the Iran National Cartographic Center, stated (https://www.entekhab.ir/fa/news/730861). Such land subsidence events and sinkholes poses significant threats to both the aquifers and the country's infrastructre. Additionally, one of the most evident casualties of the declining aquifers is undoubtedly Iran's food security, as approximately 90% of the country's strategic farmlands heavily rely on groundwater resources for support. Inland migrations, particularly in the eastern border areas of the country, which have profound implications for national security, exacerbated unemployment crisis, local disputes, frequent occurences of dust storms, floods, fires, and desrtifications are only some deep consequences of groundwater crisis in Iran.

Given the unsuccessful experiences of water management in Iran, we believe that the recovery of Iran's groundwater resources requires the implementation of a robust governance structure, which has been notably absent in the country's water management. The ill-designed governance of hydro-environmental resources makes it challenging to restore groundwater resources through the reduction of abstraction and the implementation of efficient artificial recharge methods. Change of policy approach from top-down governance structure with a cooperative and bottom-up approach that considers the interests of local stakeholders is necessary to improve the groundwater depletion problem in Iran.

Note S2

Iranian people were pioneers in systematic groundwater abstraction using the ancient qanat, a three millennia-old underground aqueduct that harnesses the force of gravity to bring groundwater to the earth's surface[5]. Ancient Qantas, recognized as UNESCO World Cultural Heritage sites, and natural springs played a dominant role in groundwater abstraction to support human life in Iran until the 1940s. Given the technological progresses and agricultural modernization, the qanats have been gradually replaced with (semi)deep wells, especially from the 1960s. The first modern study of the country's groundwater resources dates back to 1955 in the Foumanaat plain, located in the north of Iran. Subsequently, groundwater resources have garnered increasing attention, leading to widespread studies primarily focused on monitoring groundwater levels in the aquifers. Nevertheless, there are no groundwater data available until 1971 when the first groundwater monitoring network was established in the Ardabil's alluvial aquifer in the north of Iran. From that point, the monitoring network has been gradually expanding to cover almost all aquifers across the country (Fig. 4).

As shown in Fig. 4, the evolution of Iran's groundwater monitoring network, as well as the number and area of aquifers included, can be classified into three distinct evolutionary stages as described below:

(i). 1971 – 1981: The information available from the country's groundwater monitoring network during this period is notably incomplete. The network only covers approximately 2% of the aquifers (14 aquifers) with a total area of about 14,000 out of 272,000 km^2 (about 5% of the total aquifers' area in Iran) by the end of the period. Therefore, these data are not representative of the entire aquifer system, and any time series analysis using the data is valid only at the level of those aquifers.

(ii). 1981 – 2002: Iran's groundwater monitoring network has gradually developed and includes 425 aquifers (more than 53% of national aquifers) which cover 211,500 out of 272,000 km^2 of the aquifers' area in the country (more than 78% of the area of aquifers) by the end of this period. Given the good coverage of the groundwater monitoring network, the time series analysis using the produced data are valid at the level of those aquifers, and may also done with cautious for thirty secondary water basins, and even six primary water basins. Still these data are not a good representative of the whole aquifers across the country due to weak coverage of the monitoring network at the beginning of the period.

(iii). 2002 – 2017: The groundwater monitoring network includes 667 aquifers (more than 83% of national aquifers) which cover 261,500 out of 272,000 km^2 of the aquifers' area in the country (more than 96% of the area of aquifers), by the end of this period. Given the good coverage of the aquifers area in the country at the beginning (about 78%) and at the end (about 96%) of the period, the time series analysis using the produced data are valid at the level of those aquifers, thirty basins, six primary basins, and even across Iran.

Here, the comprehensive database of Iran's groundwater resources established from the water year of 2002 to 2017 was used to estimate the nationwide groundwater recharge. The groundwater monitoring network has been created in 492 out of 609 plains (Fig. S3), including 667 aquifers (Fig. S4) with 15,266 piezometers (Fig. S4) of which 12,293 are active and measure the groundwater level at monthly time scale interval. Note that some plains do not include aquifer and some others have more than one aquifer. However, in desirable conditions for coverage of Iranian aquifers, there should be 15,997 active piezometers, which will require drilling 3,704 new piezometers. The total area of aquifers is around 272,000 km^2 and the monitoring networks covered more than 96% of this area (i.e., 261,000 km^2) by the end of 2017. Therefore, the national monitoring network produces a rich groundwater data during the 2002-2017, which can properly represent the state of groundwater across the country. However, the rest of the area (i.e., about 11,000 km^2) includes 133 relatively small and less important aquifers distributed across the country.

Note S3

Here, the representative hydrograph, which showed the annual change in groundwater storage (ΔS), was calculated for all 666 aquifers in our database. ΔS was estimated by considering the aquifer area (A), specific yield (δ) and the annual average change in groundwater level (Δh) (Fig. S5). These raw data are publicly available via Data Archive of the Iran Water Resources Management Company (IWRMC) at https://stu.wrm.ir/register.asp. Given the large number of aquifers investigated in this study (i.e., 666), we aggregated the time series of groundwater storage for each hydrological basin and supplemented these data to in https://zenodo.org/record/8382150. We also reposited the specific yield (δ), and shapefiles of aquifers, basins, and primary basins in https://zenodo.org/record/8382150. Q_{off} data are usually measured for plains and basins (not aquifers) in Iran. Therefore, the aggregated Q_{off} data for each basin were supplemented in https://zenodo.org/record/8382150.

We did not consider the Q_{off} data from adjacent aquifers since the outgoing lateral fluxes from the adjacent aquifers were not available. Incoming lateral fluxes from the adjacent aquifers, artificial recharge, and natural recharge (e.g., precipitation, seepage of surface water into aquifers) are represented in the the estimated recharge itself.

References

1. Noori, R. et al. Anthropogenic depletion of Iran's aquifers. Proc. Natl Acad. Sci. USA **118**, e2024221118 (2021).
2. IPRC (Iranian Parliament Research Center). Challenges of unsustainable groundwater withdrawal in Iran: Current situation and land subsidence crisis (accessed 08 June 2023) https://rc.majlis.ir/fa/report/show/1773166 (2023).
3. Shami, S., Azar, M.K., Nilfouroushan, F., Salimi, M. & Reshadi, M.A.M. Assessments of ground subsidence along the railway in the Kashan plain, Iran, using Sentinel-1 data and NSBAS algorithm. *Int. J. Appl. Earth Obs. Geoinf.* **112**, 102898 (2022).
4. Moosavinasab, Z. & Safikhani, E. Occurrence of a sinkhole in Eaj Plain, Iran: an implication of the combined effect of karstification and reduction of groundwater reservoir in carbonate rocks. *Carbonates Evaporites* **38**, 49 (2023).
5. Maghrebi, M. et al. Anthropogenic decline of ancient, sustainable water systems: qanats. *Groundwater* **61**, 139-146 (2023).

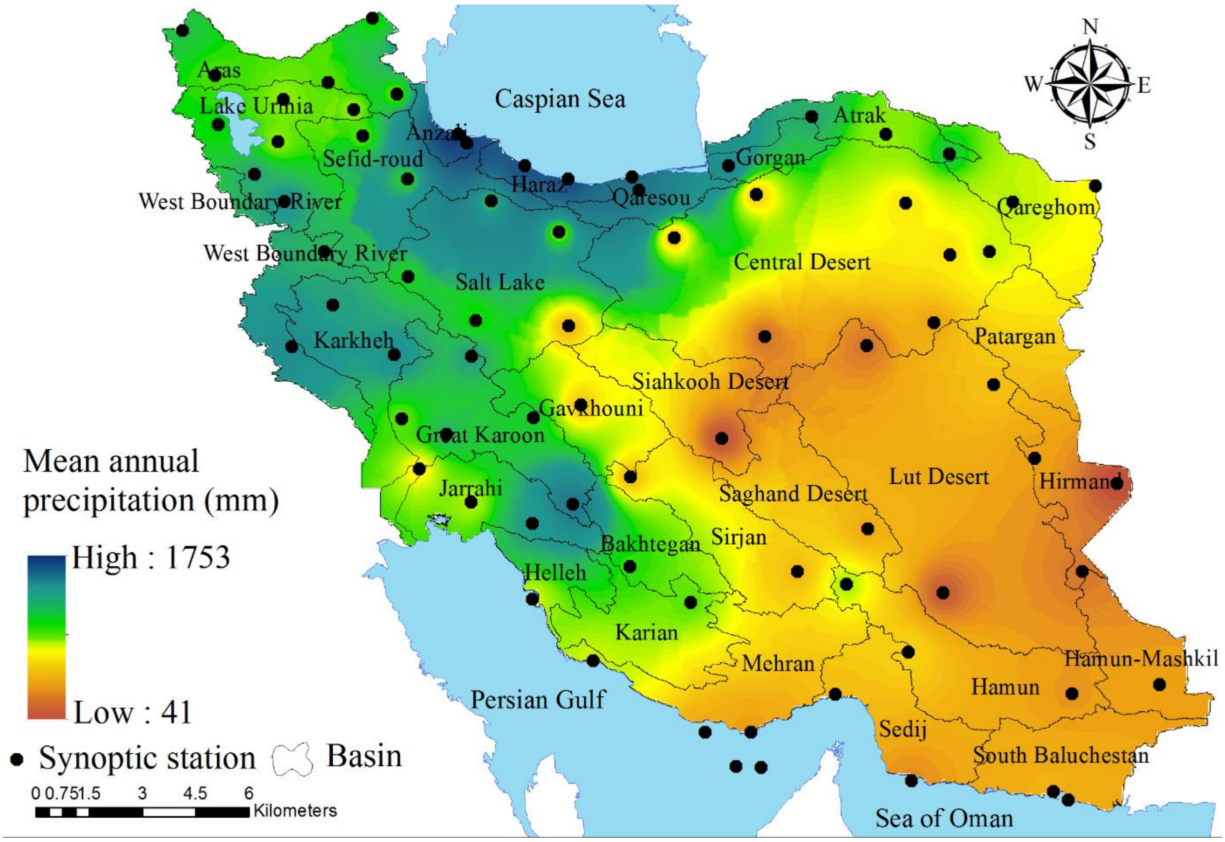

Figure S1: Hydrological basins and mean annual precipitation (mm) across Iran during 2000 to 2018 period. Hydrological basins are: Salt Lake (Daryacheh Namak), Bakhtegan, Gavkhouni (Gavkhuni), Sirjan, Hamun, Hamun-Mashkil (Hamun Mashkil), Central Desert, Lut Desert, Siahkooh Deseart, Saghand Desert, West Boundary River, Karkheh, Great Karoon (Karun or Karoun), Jarrahi (Jarahi), Helleh, Karian, Mehran, Sedij, South Baluchestan, Aras, Sefid-roud, Haraz, Qaresou, Gorgan, Atrak, Patargan, Hirmand, Lake Urmia, and Qareghom.

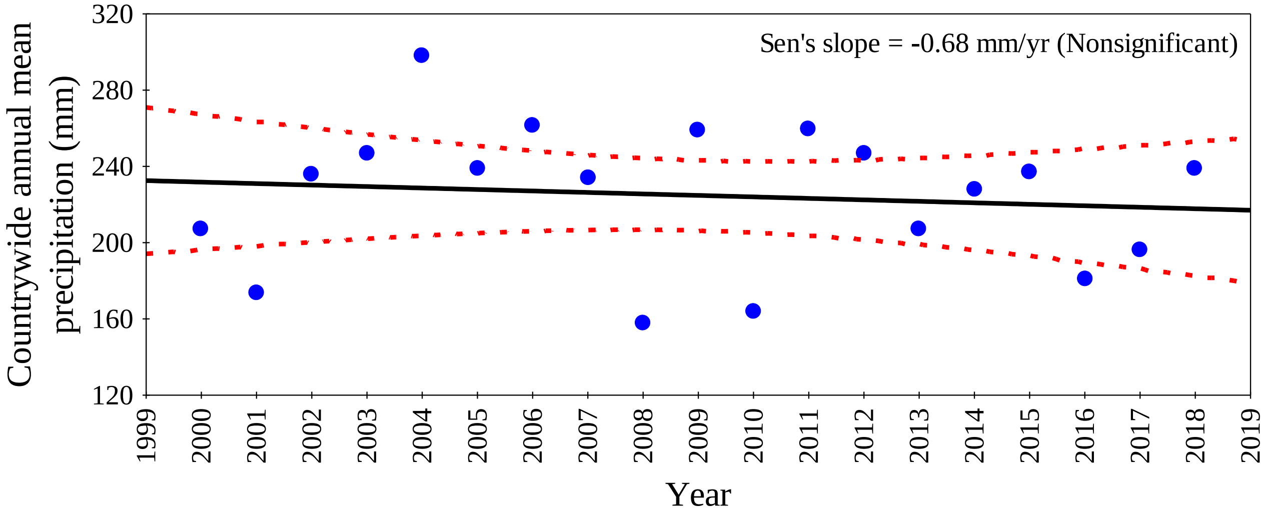

(A)

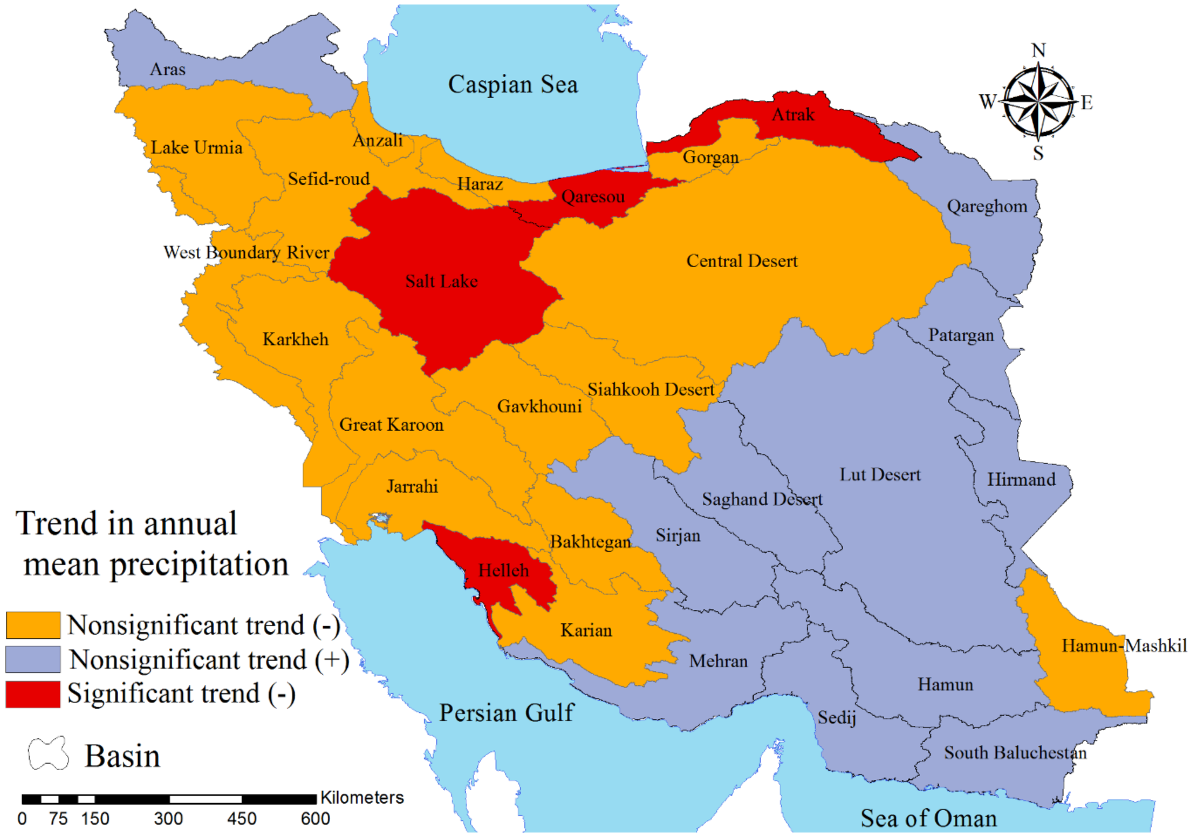

(B)

Figure S2. (A) Temporal trend of annual mean precipitation (mm) in Iran from 2000 to 2018, and (B) trend in annual mean precipitation in Iranian basins (2000-2018).

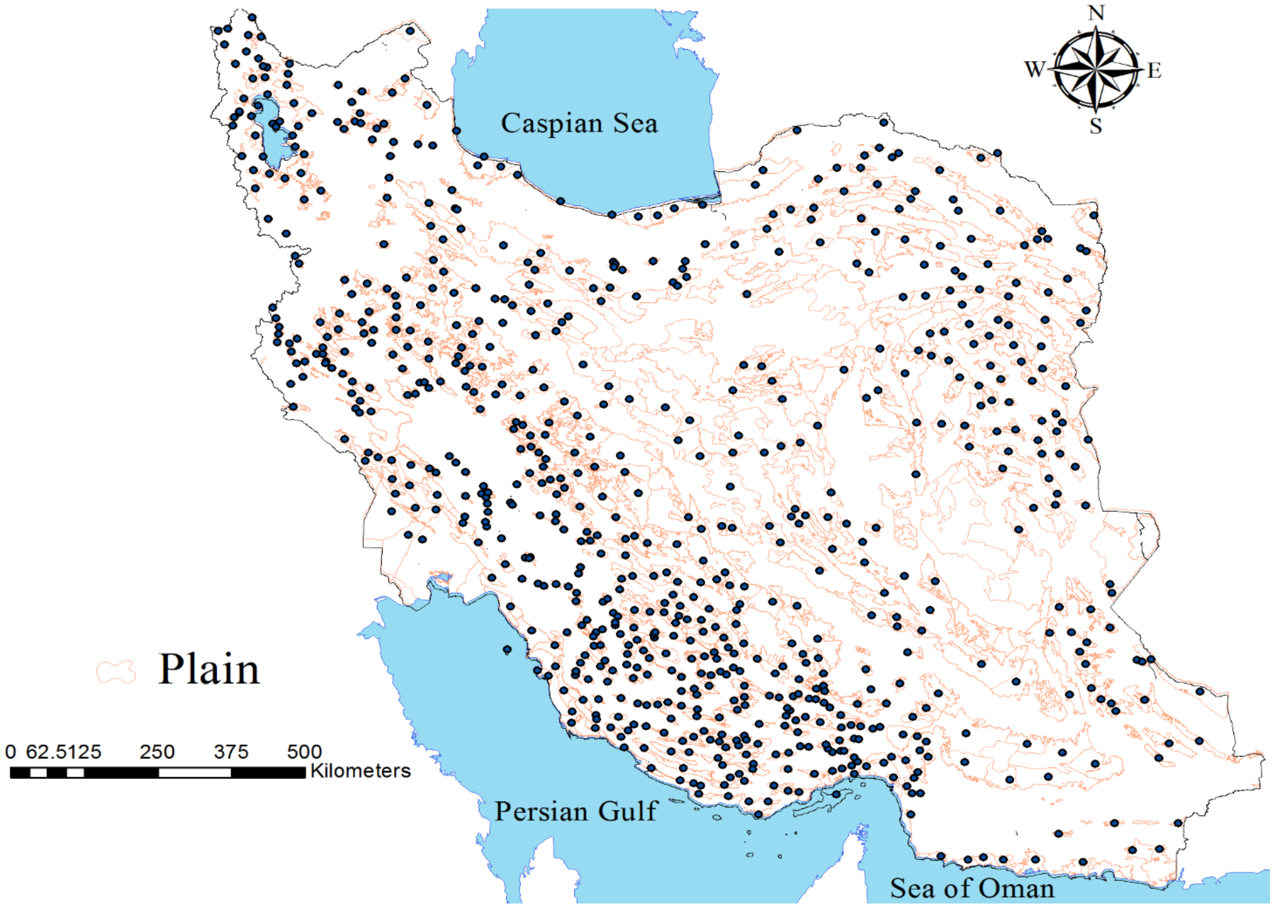

Figure S3. Distribution of Iranian plains (609 plain) across the country. The plains with active groundwater monitoring network include a dark circle in their center (492 plains).

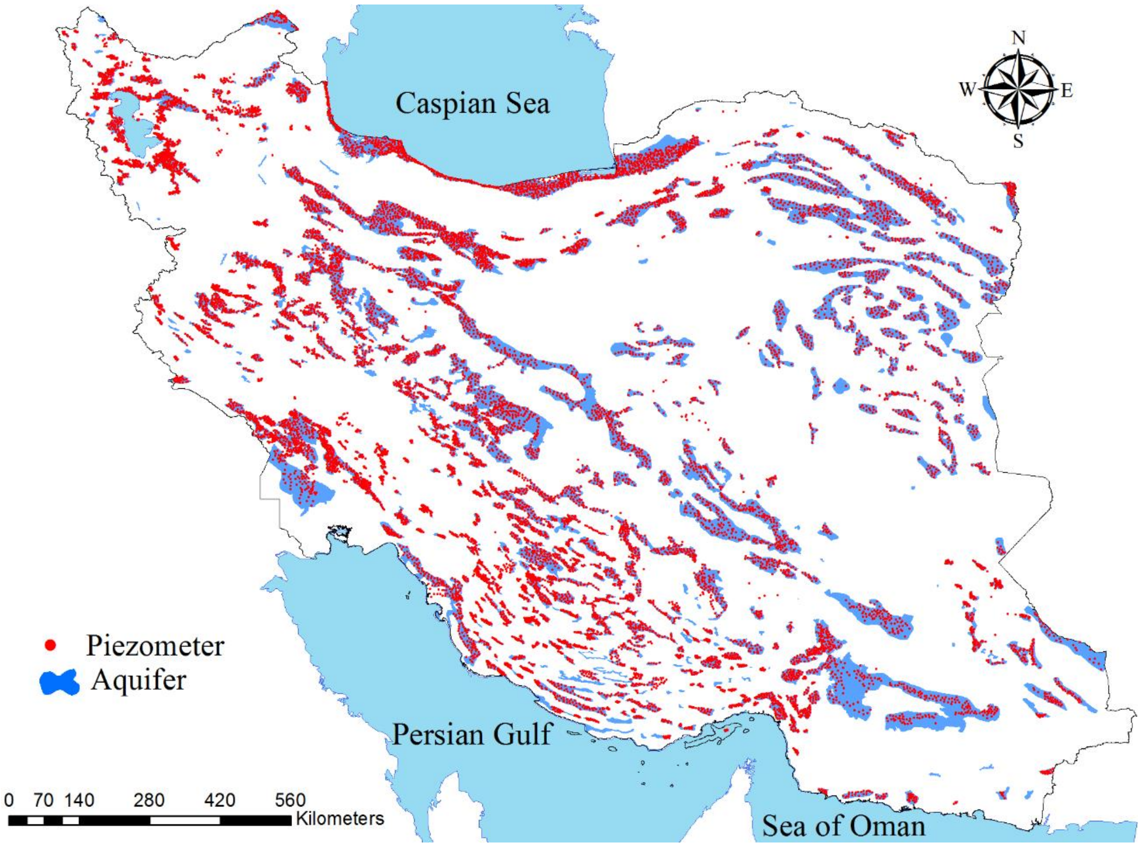

Figure S4. Distribution of Iranian aquifers (668 aquifers) and 12,293 active piezometers across the country. The total area of aquifers is around 272,000 km^2. Around 96% of aquifers' area was covered by groundwater monitoring networks by the end of 2017. Piezometers are measuring the groundwater level at monthly time scale interval.

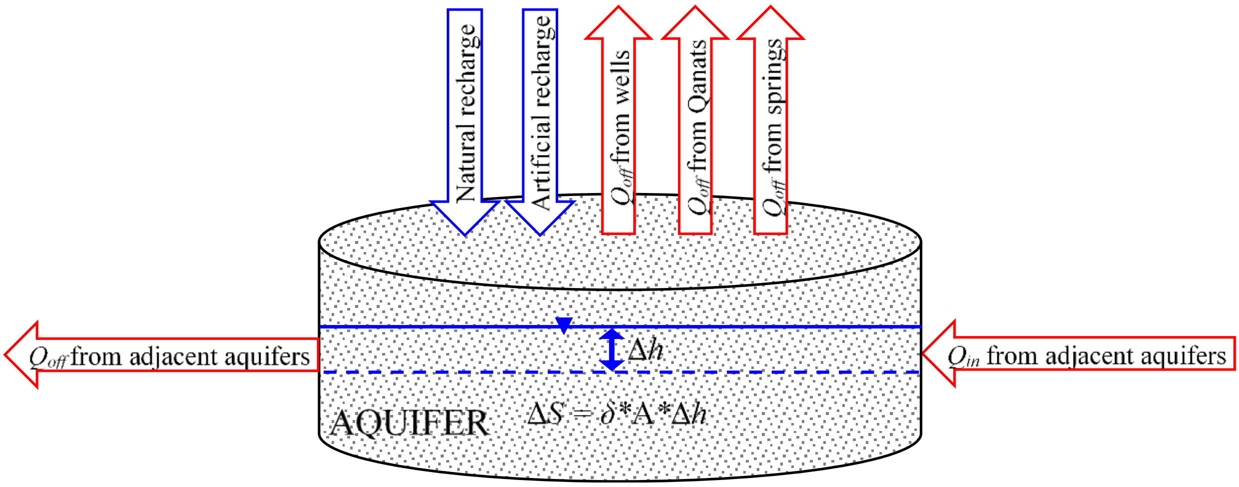

Figure S5: A shematic view of the inputs-outputs to an aquifer. Q_{off} from wells, qanats and springs, annual change in groundwater level (Δh), aquifer area (A), and specific yield (δ) are avialable thorough the national monitoring groundwater network for all 667 studied aquifers. ΔS is groundwater storage change.
